# Prognostic Significance of S100A4 in Ovarian Clear Cell Carcinoma: Its Relation to Tumor Progression and Chemoresistance

**DOI:** 10.3390/cancers17020184

**Published:** 2025-01-08

**Authors:** Misato Hayashi, Ako Yokoi, Mayu Nakagawa, Miki Hashimura, Yasuko Oguri, Makoto Saegusa

**Affiliations:** Department of Pathology, Kitasato University School of Medicine, 1-15-1 Kitasato, Minami-ku, Sagamihara 252-0374, Japan; hayashi.misato@st.kitasato-u.ac.jp (M.H.); ayokoi@med.kitasato-u.ac.jp (A.Y.); 19930216.mayu@gmail.com (M.N.); mhashimu@med.kitasato-u.ac.jp (M.H.); oguriy@med.kitasato-u.ac.jp (Y.O.)

**Keywords:** S100A4, ovarian clear cell carcinoma, epithelial–mesenchymal transition, cancer stem cell, apoptosis

## Abstract

Background/Objectives: S100A4, a small calcium-binding protein, promotes metastasis in a variety of human malignancies, but little is known about its involvement in ovarian clear cell carcinoma (OCCC). Herein, we characterized the functional role of S100A4 in this tumor type. Methods: We analyzed immunohistochemical sections from 120 OCCC patients. OCCC cell lines in which S100A4 was knocked out (KO) or overexpressed were also used to study the protein’s effects. Results: Stable overexpression of S100A4 decreased the proliferation of OCCC cell lines (concomitant with more cells in G1 and fewer in the G2/M phase of the cell cycle). S100A4 overexpression also reduced susceptibility to cisplatin-induced apoptosis (probably due to an increased BCL2: BAX ratio), accelerated epithelial–mesenchymal transition (EMT)-related cell mobility, and enhanced cancer stem cell (CSC) properties (including increases in both spheroid formation and in the aldehyde dehydrogenase 1 (ALDH1)^high^ population). In contrast, S100A4 KO generally induced the opposite phenotypes, although it did not affect migration capability. In clinical OCCC samples, high S100A4 expression was associated with a low frequency of cleaved poly-(ADP-ribose) polymerase 1-positive apoptotic cells, a reduced proliferative rate, and expression of high ALDH1 and vimentin levels. In addition, a high S100A4 score was a significant (but not independent) prognostic factor in OCCC. Conclusions: Our findings suggest that S100A4 may be an unfavorable prognostic factor in OCCC, as it accelerates tumor progression and promotes chemoresistance through the modulation of proliferation, susceptibility to apoptosis, and EMT/CSC properties.

## Simple Summary

Ovarian clear cell carcinoma (OCCC) is a distinct subtype of epithelial ovarian carcinoma in terms of clinical, histopathological, and genetic features. The prognosis of patients with advanced OCCC is extremely poor; this is probably due to the inherent chemoresistance of this cancer. S100A4, a member of the S100 protein family, lacks enzymatic activity and instead exerts its biological effects through interaction with target proteins. S100A4 facilitates the development of highly aggressive tumors and formation of metastasis in a variety of human malignancies. Here, we hypothesized that S100A4 might have prognostic significance in OCCC through the modulation of multiple tumor biological functions. Our findings suggest that S100A4 accelerates tumor progression and promotes chemoresistance through the modulation of proliferation, susceptibility to apoptosis, and epithelial–mesenchymal transition/cancer stem cell properties. The potential for S100A4 to confer chemoresistance highlights its pivotal role in the clinical course of OCCC.

## 1. Introduction

Epithelial ovarian carcinoma (EOC) is the seventh most commonly diagnosed carcinoma among women worldwide and has the highest mortality rate of all gynecological carcinomas [1,2]. The 5-year overall survival is below 45% and it decreases to 25% among patients with advanced EOC [1]. The dismal survival rate in advanced EOC patients is due to tumor recurrence within 2 years of diagnosis, even after primary debulking surgery and adjuvant chemotherapy [3]. Recurrent tumors often develop resistance to conventional chemotherapy; this in turn results in a very poor survival rate and accounts for the high lethality of EOC [4].

Ovarian clear cell carcinoma (OCCC) is a distinct subtype of EOC in terms of clinical, histopathological, and genetic features. Its prevalence is dependent on geographical location; OCCC accounts for 5–10% of all EOC in North America and 12% in other Western countries but constitutes a higher fraction of EOC in East Asia, where it accounts for 20–30% and 10–12% of cases in Japan and Korea, respectively [5,6,7]. Unlike other EOC types, endometriosis is a recognized risk factor for OCCC, as women with this condition are three times more likely to develop this malignancy when compared to those without endometriosis [8,9]. Patients with OCCC tend to be diagnosed at younger ages and at an earlier stage, and often suffer from thromboembolic vascular complications [10,11]. Consistent with the aggressive clinical course, the prognosis of patients with advanced OCCC is extremely poor; this is probably due to the inherent chemoresistance of this cancer [12].

S100A4, a member of the S100 protein family, is a calcium-binding protein with two EF hand domains [13,14,15]. The protein lacks enzymatic activity and instead exerts its biological effects through interaction with target proteins [13,14,15]. Although S100A4 facilitates the development of highly aggressive tumors and formation of metastasis in a variety of human malignancies, including ovarian carcinoma and uterine carcinosarcoma [16,17,18], little is known about its functional role in OCCC.

Here, we hypothesized that S100A4 might have prognostic significance in OCCC through the modulation of multiple tumor biological functions. To test this, we looked for correlations between S100A4 expression and proliferation, susceptibility to apoptosis, mobility, and epithelial–mesenchymal transition (EMT)/cancer stem cell (CSC) features in OCCC cells. We also determined whether there were correlations between S100A4 expression and well-documented clinicopathological parameters in OCCC.

## 2. Materials and Methods

### 2.1. Plasmids and Cell Lines

Two OCCC cell lines, OVISE (RRID:CVCL_3116) and TOV-21G (RRID:CVCL_3613), were obtained from the National Institute of Biomedical Innovation (Osaka, Japan) and the American Type Culture Collection (Manassas, VA, USA), respectively. They were used within 6 months of thawing and were periodically authenticated by monitoring of cell morphology and growth curve analysis. All experiments were performed with mycoplasma-free cells.

Based on the endogenous S100A4 status, an S100A4 overexpression (OE) model was established using the OVISE cell line (OV-S100A4 OE) as described previously [17]. An S100A4 knockout (KO) cell line was also generated using TOV-21G cells (TOV-S100A4 KO). Briefly, the guide RNA sequence (gRNA: 5′-CCACAAGTACTCGGGCAAAG-3′) was designed using CRISPRdirect (https://crispr.dbcls.jp accessed on 22 November 2022) and cloned into pSpCas9n(BB)-2A-Puro (PX459) V2.0 (Addgene #62988) to establish the S100A4 KO cell line as described previously [19].

### 2.2. Clinical Cases

A total of 120 consecutive OCCC cases, surgically resected at Kitasato University Hospital between 2005 and 2019, were selected from our patient records according to the criteria of the 2014 World Health Organization classification, the TNM, and the International Federation of Gynecology and Obstetrics (FIGO) classification [20,21,22]. All patients underwent oophorectomy with or without hysterectomy. No patients had received paclitaxel/carboplatin-based chemotherapy before surgical treatment, whereas most patients had been treated with chemotherapy after surgical resection. All cases showed complete resection of the tumors, and no cases had residual tumors after debulking surgery. All tissues were routinely fixed in 10% formalin and processed for embedding in paraffin wax. Approval for this study was given by the Ethics Committee of the Kitasato University School of Medicine (B20-181: approval date, 7 October 2020).

### 2.3. Immunohistochemistry (IHC)

IHC was performed using a combination of the microwave oven heating and polymer immunocomplex (Envision, Dako, Copenhagen, Denmark) methods as described previously [16,17,19].

For evaluation of IHC findings, scores for S100A4, ALDH1, and vimentin were derived by multiplying the percentage of immunopositive cells by the immunointensity as described previously [16,17,19]. The Ki-67 labeling indices (LIs) were derived by calculating the percentage of tumor cells that exhibited nuclear Ki-67 immunoreactivity (at least 500 tumor cells were evaluated). The number of cleaved PARP1-positive cells in five randomly selected high-power fields (HPFs) was also calculated. S100A4 scores were subdivided into high and low categories based on a mean value of 3 as the cutoff (Supplementary Figure S1).

### 2.4. Antibodies and Reagents

Anti-poly (ADP-ribose) polymerase 1 (PARP1), anti-cleaved PARP1, anti-cleaved caspase-3, anti-vimentin, and anti-phospho (p) Rb at Ser807/811 antibodies were purchased from Cell Signaling Technology (Danvers, MA, USA). Anti-S100A4 and anti-cyclin A2 antibodies were obtained from Abcam (Cambridge, MA, USA). Anti-p53, anti-BCL2, anti-p21^waf1^, anti-cyclin D1, and anti-Ki-67 antibodies were from Dako (Copenhagen, Denmark). Anti-p27^kip1^, anti-BAX, anti-Rb, anti-aldehyde dehydrogenase 1 (ALDH1), and anti-X-linked inhibitor of apoptosis protein (XIAP) antibodies were from BD Biosciences (San Jose, CA, USA). Anti-β-actin and anti-myosin 9 (MYH9) were from Sigma-Aldrich Chemicals (St. Louis, MO, USA) and Proteintech (Rosemont, IL, USA), respectively. Cisplatin (CDDP; P4394) was also obtained from Sigma-Aldrich Chemicals.

### 2.5. Western Blot and Co-Immunoprecipitation (Co-IP) Assays

Extraction of total cellular proteins, Western blot, and co-IP assays were carried out as described previously [16,17,19]. The signals were analyzed using ImageJ software version 1.41 (NIH, Bethesda, MD, USA; http//imageJ.nih.gov/ij accessed on 10 September 2020) as described previously [16,17,19]. The reconstructed images of all blots with membrane edges visible are accessible in the Supplementary Materials, because some of the original full-length blots were cut prior to hybridization with antibodies.

### 2.6. Flow Cytometry and Aldefluor Assay

Cells were stained with propidium iodide (Sigma) for cell cycle analysis or subjected to a fluorogenic dye-based Aldefluor assay (Stem Cell Technologies, Grenoble, France) to measure ALDH1 enzyme activity. Flow cytometric analysis using a BD FACS Calibur (BD Biosciences) and CellQuest Pro software version 3.3 (BD Biosciences) was then performed as described previously [16,17].

### 2.7. Spheroid Assay

Cells (×10^3^) were plated in low cell binding plates (Thermo Fisher Scientific, Yokohama, Japan) in Cancer Stem Cell Premium (ProMab Biotech, Richmond, CA, USA). Uniform spheroids of at least 50 μm in diameter were counted approximately two weeks after plating as described previously [16,17].

### 2.8. Cell Counting Kit-8 Assay

Cell viability after ADR treatment was evaluated using the Cell Counting Kit-8 (CCK-8; Dojindo Lab, Kumamoto, Japan) as described previously [23].

### 2.9. Apoptotic Index

The number of apoptotic cells identified in HE-stained sections was calculated by counting the mean number of apoptotic figures per field as described previously [23].

### 2.10. Wound Healing Assay

After a cell monolayer formed, the area of the scratch wound, which was created with a sterile 200 μL tip, was calculated in pixels as wound closure using ImageJ software version 1.41 as described previously [16,17,19].

### 2.11. Migration Assay

Cell migration was determined using 24-well Transwell chambers with 8 μm pore size (Corning, NY, USA). After 24 h, the number of migrated cells stained by hematoxylin–eosin (HE) on the bottom surface of the polycarbonate membranes was counted visually using a light microscope as described previously [16,17,19].

### 2.12. Statistics

Sample size calculations were guided by the previous literature [24,25].

Comparisons between groups of data were performed using the Mann–Whitney *U*-test or Chi-square test, as appropriate. For all figures, experiments were performed in triplicate, and data are presented as mean ± SD. Overall survival (OS) and progression-free survival (PFS) were estimated using Kaplan–Meier methods, and the statistical comparisons were made using the logrank test. Univariate and multivariate analyses were also performed using the Cox proportional hazards regression model. The cutoff for statistical significance was set as *p* < 0.05.

## 3. Results

### 3.1. S100A4 Suppresses Both Proliferation and Apoptosis in OCCC Cells

To examine the functional role of S100A4 in OCCC cells, we generated three independent TOV-S100A4 KO cell line clones (KO#17, KO#25, and KO#35) and two independent OV-S100A4 OE cell lines (OE#13 and OE#27) (Supplementary Figure S2).

**Figure 1:**
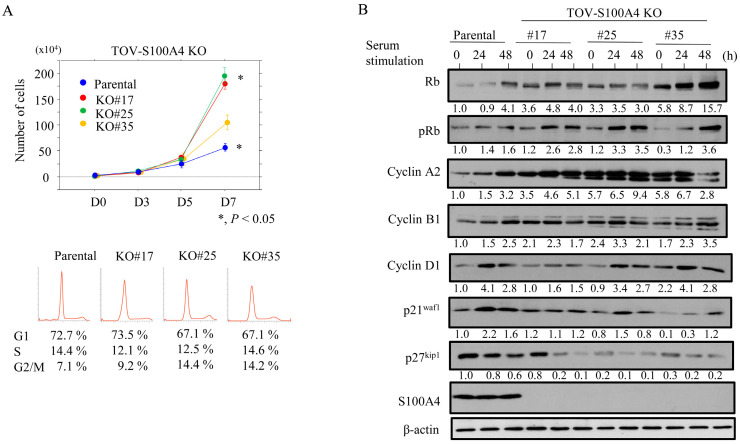
Changes in proliferation following S100A4 knockout in OCCC cells. (**A**) Upper: S100A4 KO and parental cells were seeded at low density. P0, P3, P5, and P7 are 0, 3, 5, and 7 days after seeding, respectively. Lower: flow cytometry analysis of S100A4 KO and parental cells 3 days after seeding (P3). (**B**) Western blot analysis for the indicated proteins in total lysates from S100A4 KO and parental cells following re-stimulation of serum-starved (24 h) cells with 10% serum for the indicated times. Each signal intensity (calculated by normalization to β-actin) is indicated in lower sites of the panels. Expression levels in the absence of re-stimulation (0 h) in parental cells were set as 1.

**Figure 2:**
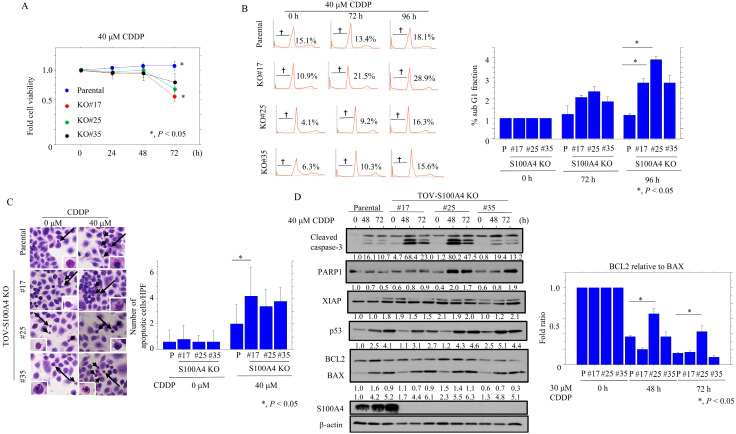
Changes in apoptosis following S100A4 knockout in OCCC cells. (**A**) Treatment of S100A4 KO and parental cells (P) with 40 μM CDDP for 24 h. Cell viability was measured using the CCK-8 Viability kit. The absence of CDDP treatment is set as 1. (**B**) Left: flow cytometric cell cycle analysis for S100A4 KO and parental cells (P) after 40 μM CDDP treatment for the time shown. † Daggers indicate sub-G1 fractions. Right: the percentages of cells undergoing apoptosis (sub-G1) were calculated. (**C**) Left: S100A4-KO and parental cells (P) undergoing apoptosis after 40 μM CDDP treatment are indicated by arrows. The closed boxes in the right-hand panels are magnified in the insets. Original magnification, ×200 and ×400 (insets). Right: numbers of apoptotic cells are shown as mean ± SD. (**D**) Left: Western blot analysis for the indicated proteins in total lysates from S100A4 KO and parental cells with 40 μM CDDP treatment for the time shown. Each signal intensity (calculated by normalization to β-actin) is indicated in lower sites of the panels. Expression levels in the absence of CDDP treatment (0 h) in parental cells were set as 1. Right: the BCL2: BAX ratio was calculated by normalization to β-actin using ImageJ version 1.41. Expression levels in the absence of CDDP treatment (0 h) were set as 1.

The TOV-S100A4 KO cells proliferated more rapidly than the parental cells; consistent with this, there were proportionally more cells in the G2/M phase and fewer in the G1 phase (Figure 1A). We next examined the impact of S100A4 loss on several cell cycle-related markers. The TOV-S100A4 KO cells were rendered quiescent by serum starvation prior to the re-addition of serum to trigger cell cycle entry, and samples were taken at 24 and 48 h post-serum stimulation. The levels of Rb, pRb, and cyclin A2 were higher in the TOV-S100A4 KO cells when compared to the parental cells, whereas the expression of both p21^waf1^ and p27^kip1^ was lower in the KO cells (Figure 1B and Supplementary Figure S3A). The TOV-S100A4 KO cells were also significantly more sensitive to CDDP treatment than the parental cells (Figure 2A) and there was a significant increase in the sub-G1 fraction (Figure 2B) and number of apoptotic cells in the CDDP-treated KO cells (Figure 2C). These data are consistent with the increased expression of cleaved caspase-3 and PARP1. However, there were no changes in the expression of XIAP or p53, or the BCL2: BAX ratio (with the exception of KO#25) (Figure 2D and Supplementary Figure S3B), when compared to the parental cells.

**Figure 3:**
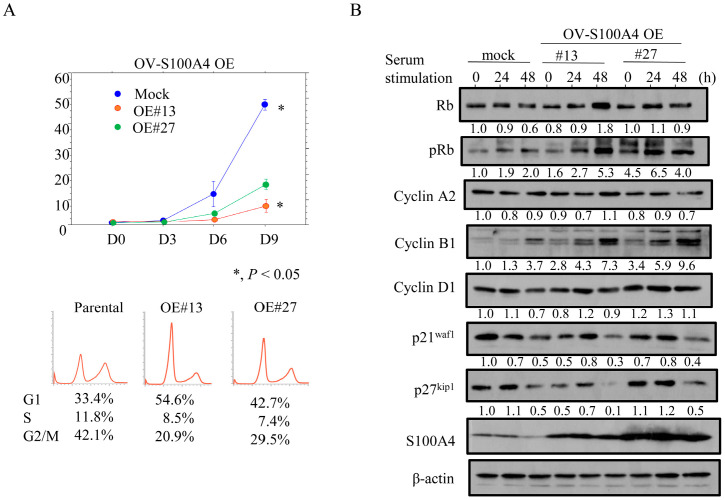
Changes in proliferation following S100A4 overexpression in OCCC cells. (**A**) Upper: S100A4 OE and mock cells were seeded at low density. P0, P3, P6, and P9 are 0, 3, 6, and 9 days after seeding, respectively. Lower: flow cytometry analysis of S100A4 OE and mock cells 3 days after seeding (P3). (**B**) Western blot analysis for the indicated proteins in total lysates from S100A4 OE and mock cells (Mo) following re-stimulation of serum-starved (24 h) cells with 10% serum for the indicated times. Each signal intensity (calculated by normalization to β-actin) is indicated in lower sites of the panels. Expression levels in the absence of re-stimulation (0 h) in mock cells were set as 1.

**Figure 4:**
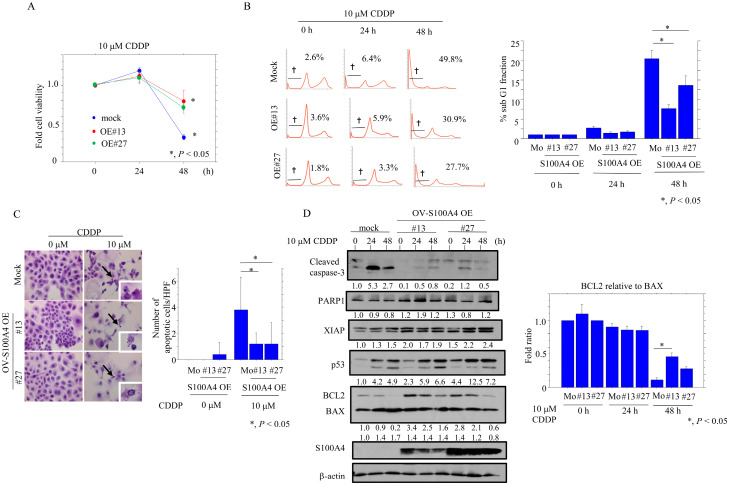
Changes in apoptosis following S100A4 overexpression in OCCC cells. (**A**) Treatment of S100A4 OE and mock cells (Mo) with 10 μM CDDP for 24 h. Cell viability was measured using the CCK-8 Viability kit. The absence of CDDP treatment is set as 1. (**B**) Left: flow cytometric cell cycle analysis for S100A4 OE and mock cells (Mo) after 10 μM CDDP treatment for the time shown. † Daggers indicate sub-G1 fractions. Right: the percentages of cells undergoing apoptosis (sub-G1) were calculated. (**C**) Left: after 10 μM CDDP treatment, S100A4 OE and mock cells (Mo) undergoing apoptosis are indicated by arrows. The closed boxes in the right panels are magnified in insets. Original magnification, ×200 and ×400 (insets). Right: the numbers of apoptotic cells are shown as mean ± SD. (**D**) Left: Western blot analysis for the indicated proteins in total lysates from S100A4 OE and mock cells with 10 μM CDDP treatment for the time shown. Each signal intensity (calculated by normalization to β-actin) is indicated in lower sites of the panels. Expression levels in the absence of CDDP treatment (0 h) in mock cells were set as 1. Right: the BCL2: BAX ratio was calculated by normalization to β-actin using ImageJ version 1.41. Expression levels in the absence of CDDP treatment (0 h) were set as 1.

In contrast, the OV-S100A4 OE cells tended to proliferate more slowly as compared to the mock cells in the exponential growth phase; there were also proportionally fewer cells in the G2/M phase and more in the G1 phase (Figure 3A), as well as increased expression of pRb and cyclin B1 (Figure 3B and Supplementary Figure S4A). The OV-S100A4 OE cells were more resistant to CDDP than the mock cells (Figure 4A); this was consistent with a reduced sub-G1 fraction (Figure 4B), a reduced number of apoptotic cells (Figure 4C), lower levels of cleaved caspase-3, and an increased BCL2: BAX ratio (Figure 4D and Supplementary Figure S4B). In the OCCC cells, we also found that p53 and MYH9 robustly interacted with S100A4, and co-immunoprecipitated with PARP1, which was used as a positive control [26] (Supplementary Figure S5).

Together, these findings suggest that S100A4 simultaneously inhibits proliferation and attenuates apoptosis in OCCC cells.

### 3.2. S100A4 Accelerates EMT-Related Mobility and Enhances CSC Properties in OCCC Cells

**Figure 5:**
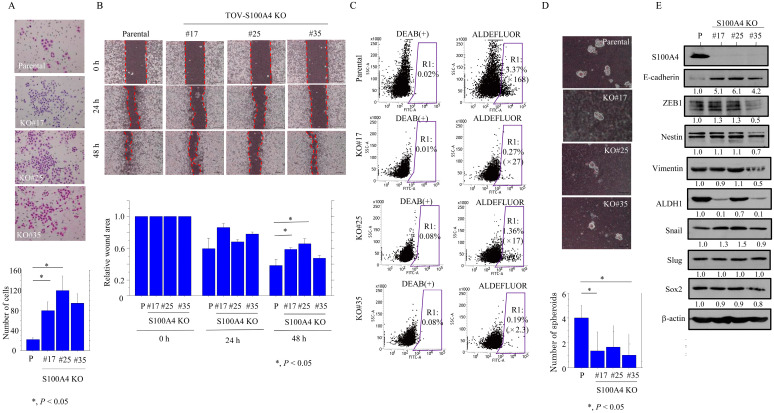
Changes in cell motility and CSC properties following S100A4 knockout in OCCC cells. (**A**) Upper: S100A4 KO and parental cells (P) were seeded in 24-well Transwell plates and incubated for 24 h in medium without serum. Cells were stained with HE and counted using a light microscope. Lower: the number of migrated cells is presented as mean ± SD. (**B**) Upper: wound healing assay with S100A4 KO and parental cells. A scratch ‘wound’ was introduced to the middle of wells containing cells grown to confluency, and phase-contrast images were taken after 24 and 48 h. Lower: the values of wound areas in 0 h were set as 1. The fold wound areas in S100A4 KO and parental cells (P) are shown as mean ± SD. (**C**) Aldefluor analysis of S100A4 KO and parental cells. ALDH1 activity-negative cells are located in the area to the far left of each plot, and the positive cells are within the black gate (R1). The percentage of live single-cell populations, as well as the values of R1 in ALDEFLUOR relative to R1 in DEAB(+) (indicated by parentheses), contained in each gate are shown. (**D**) Upper: Phase-contrast photograms of spheroids derived from S100A4 KO and parental cells (P) following 2 weeks of growth. Lower: the number of spheroids. (**E**) Western blot analysis for the indicated proteins in total lysates from S100A4 KO and parental cells (P). Each signal intensity (calculated by normalization to β-actin) is indicated in lower sites of the panels. Expression levels in parental cells were set as 1.

To examine whether S100A4 affects cell motility, we carried out migration and scratch assays. The TOV-S100A4 KO cells were significantly more mobile than the parental cells (Figure 5A) but refilled wounded empty spaces more slowly (Figure 5B).

Since S100A4 is a stemness marker expressed in normal cells [27], we examined whether there was an association between S100A4 and CSC properties. The Aldefluor assay revealed a significant decrease in the ALDH1^high^ activity population in the TOV-S100A4 KO cells as compared to the parental cells (Figure 5C). This was consistent with a lower number of well-defined, round TOV-S100A4 KO spheroids that were over 50 μm in size (Figure 5D). There were no significant changes in the expression of several EMT/CSC-related markers (Figure 5E and Supplementary Figure S6A).

**Figure 6:**
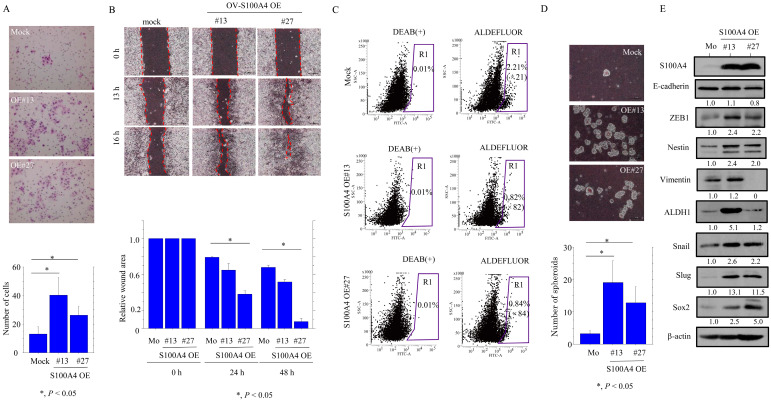
Changes in cell motility and CSC properties following S100A4 overexpression in OCCC cells. (**A**) Upper: S100A4 OE and mock cells were seeded in 24-well Transwell plates and incubated for 24 h in medium without serum. Cells were stained with HE and counted using a light microscope. Lower: the number of migrated cells is presented as mean ± SD. (**B**) Upper: wound healing assay with S100A4 OE and mock cells. A scratch ‘wound’ was introduced to the middle of wells containing cells grown to confluency, and phase-contrast images were taken after 24 and 48 h. Lower: the values of wound areas in 0 h were set as 1. The fold wound areas in S100A4 OE and mock cells (Mo) are shown as mean ± SD. (**C**) Aldefluor analysis of S100A4 OE and mock cells. Cells negative for ALDH1 activity are located in the area to the far left of each plot, and the positive cells are within the black gate (R1). The percentage of live single-cell populations, as well as the values of R1 in ALDEFLUOR relative to R1 in DEAB(+) (indicated by parenthesis), contained in each gate are shown. (**D**) Upper: Phase-contrast photograms of spheroids derived from S100A4 OE and mock cells (Mo) following 2 weeks of growth. Lower: the number of spheroids. (**E**) Western blot analysis and the indicated proteins in total lysates from S100A4 OE and mock cells (Mo). Each signal intensity (calculated by normalization to β-actin) is indicated in lower sites of the panels. Expression levels in mock cells were set as 1.

In contrast to the TOV-S100A4 KO cells, the OV-S100A4 OE cells exhibited enhanced migration rates (Figure 6A) and movement (Figure 6B); they also displayed CSC properties with a significant increase in the fraction of the ALDH^high^ population (Figure 6C) and a greater number of large, well-defined spheroids (Figure 6D) as compared to the mock cells. These phenotypes were consistent with the increased expression of EMT/CSC-related markers including ZEB1, Nestin, Snail, Slug, and Sox2 but not E-cadherin (Figure 6E and Supplementary Figure S6B).

These findings suggest that S100A4 increases EMT-related motility and CSC properties in OCCC cells.

### 3.3. Prognostic Significance of S100A4 in OCCC

**Figure 7:**
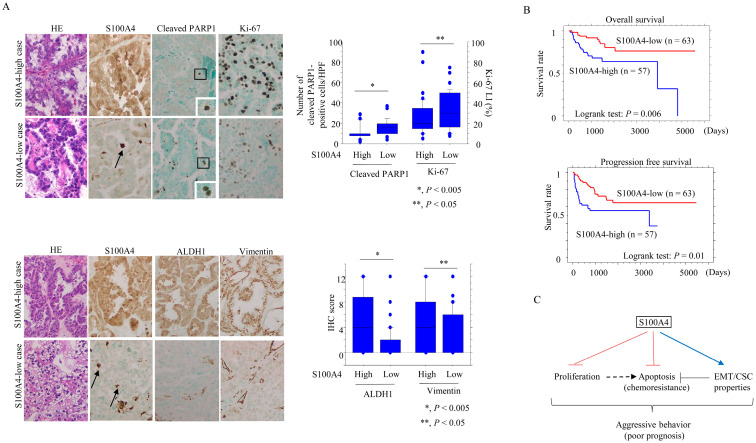
Relationship between S100A4 expression, proliferation, apoptosis, and EMT/CSC properties in OCCC tissues. (**A**) Upper- and lower-left: staining with HE and IHC for the indicated proteins in samples with low and high S100A expression. The closed boxes are magnified in the insets of the cleaved PARP1 panels. Stromal S100A4-positve lymphocytes are indicated by arrows. Original magnification, ×200 and ×400 (insets). Upper- and lower-right: number of cleaved PARP1-positive cells and Ki-67 LIs (upper) and IHC scores for ALDH1 and vimentin (lower) in the S100A4-high and -low categories. The number, LIs, and scores shown are mean ± SD. (**B**) OS (**upper**) and PFS (**lower**) relative to S100A4 (low versus high expression) in OCCC. n, number of cases. (**C**) Schematic representation of the functional roles of S100A4 during OCCC progression. S100A4 expression contributes to tumor progression through modulation of proliferation, susceptibility to apoptosis, and EMT/CSC properties; this in turn results in aggressive behavior and a poor prognosis.

We next compared the immunoreactivities of cytoplasmic and nuclear S100A4 with those of nuclear cleaved PARP1 (a marker of apoptosis) and Ki-67 (a marker of proliferation), cytoplasmic ALDH1 (a CSC marker), and vimentin (an EMT marker). The number of S100A4-positive stromal infiltrating lymphocytes is also shown in Figure 7A. High S100A4 scores were significantly associated with reduced numbers of cleaved PARP1-positive cells and lower Ki-67 LIs, but with higher numbers of ALDH1- and vimentin-positive cells (Figure 7A).

A high S100A4 score was associated with advanced clinical stage, higher pT factor, distant metastasis, and dissemination (Supplementary Table S1); this was consistent with worse OS and PFS in patients with S100A4-high tumors (Figure 7B). Comparison of univariate and multivariate Cox analyses revealed that S100A was a significant (but not independent) prognostic indicator for both OS and PFS (Table 1).

Consistent with our OCCC cell model data, this analysis of clinical samples confirms that high S100A4 expression may be an unfavorable prognostic factor in OCCC due to its effects on the proliferation, apoptosis, and expression of EMT/CSC-related markers.

## 4. Discussion

The present study clearly provides evidence that TOV-S100A4 knockout engenders increased proliferation that is concomitant with increased Rb and cyclin A2 expression and decreased p27^kip1^ expression. In contrast, S100A4 overexpression suppresses proliferation and increases the levels of pRb and cyclin B1. These results are consistent with the inverse correlation between the S100A4 score and Ki-67 LIs that we observed in OCCC tissues and in endometrial carcinoma (Em Ca) cells in our previous study [17].

While one previous study found that siRNA-mediated knockdown of S100A4 reduced proliferation in prostate and Em Ca cells [28,29], this was not evident in another study that focused on Em Ca cells [30]. This discrepancy may be due to cell- and tissue-specific factors that modulate the effect of S100A4 on cellular proliferation. For example, S100A4 facilitates cyclin B1 relocalization to spindle pole areas prior to cyclin B1-Cdk1 activation [31], and perhaps this is a context-specific effect.

We also found that S100A4 knockout sensitized OCCC cells to CDDP-induced apoptosis, whereas S100A4 overexpression conferred resistance. This is consistent with the inverse correlation between S100A4 levels and the number of cleaved PARP1-positive cells in OCCC tissues. Since cells are more sensitive to radiation during the G2/M phase [32], and S100A4 knockout leads to an accumulation of cells in G2/M, we suggest that S100A4-dependent alterations in cell cycle progression may underlie the sensitivity to genotoxic agents in OCCC cells.

In addition to its effects on the cell cycle, S100A4 suppresses apoptosis by sequestering and inactivating p53, which blocks transactivation of pro-apoptotic *BAX* [33]. Consistent with this, we show that S100A4 overexpression reduces BAX expression and increases BCL2 expression. This effectively increases the BCL2: BAX ratio, which in turn may suppress mitochondria-dependent apoptosis. In addition, S100A4 knockdown induces apoptosis through the activation of caspase-3, caspase-9, cleavage of PARP, and release of cytochrome c into the cytosol [34].

We also show that S100A4 overexpression enhances EMT-related cell mobility and migration and confers CSC-like phenotypes in OCCC cells. In addition, S100A4 expression was positively associated with the expression of EMT/CSC-related markers in OCCC tissues. Given that EMT generates cells with CSC-like features [35], we suggest that S100A4 may promote OCCC ‘stemness’ through its ability to increase EMT-related mobility. S100A4 overexpression suppressed proliferation yet increased motility, whereas S100A4 KO elicited the opposite effects. This is consistent with findings that the proliferation and migration of tumor cells may be mutually exclusive phenotypes [30]. The expression of E-cadherin was not affected by either S100A4 overexpression or knockout. This lack of correlation may be due to the association of cancer stemness with a partial EMT phenotype rather than full-blown EMT (a state also referred to as CSC plasticity) [36,37,38].

In contrast to S100A4 overexpression, S100A4 knockout increased proliferation, sensitized to apoptosis, and enhanced cell mobility. Despite the increased cell mobility, S100A4 knockout actually reduced the cellular migratory capability. We suggest that this discrepancy may be since these two phenotypes are independently regulated by different signal transduction pathways. Further studies to address this point are clearly warranted.

We have previously demonstrated that, in ovarian high-grade serous carcinoma, overexpression of S100A4 induces EMT and CSC properties, alters cell proliferation, apoptosis, and migration capability, and regulates Snail expression, through the interaction with p53 and MYH9 [16]. Our present data showing interactions between S100A4, MYH9, and p53 in OCCC cells suggest that similar mechanisms may also operate in OCCC cells.

Finally, we found high S100A4 expression in OCCC patient samples was associated with aggressive features and a poor prognosis. We infer that the extremely poor response rate (20–50%) to platinum-based chemotherapy in advanced OCCC [39] may in part be due to S100A4 overexpression. 

## 5. Conclusions

Together with its effects on tumor progression and EMT/CSC phenotypes, the potential for S100A4 to confer chemoresistance highlights its pivotal role in the clinical course of OCCC (Figure 7C).

## Figures and Tables

**Table 1:** Univariate and multivariate analyses for overall survival and progression-free survival in ovarian clear cell carcinoma.

Univariate Analysis					Multivariate Analysis				
Variables	Cutoff	Logrank c2	*p*-value	Unfavorabel Factor	Variable	Cutoff	Hazard Ratio	95% CI	*p*-value
Overall survival					Overall survival				
S100A4 score	2/3	7.53	0.006	High score	S100A4 score	2/3	1.5	0.54–3.96	0.4
Age (years)	59/60	0.37	0.5		FIGO stage	I/II·III·V	0.3	0.03–2.14	0.2
FIGO stage	I/II·III·V	29.2	<0.0001	II·III·IV	LN metastasis	−/+	1.0	0.22–4.71	1
LN metastasis	−/+	5.62	0.01	+	Distant metastasis	−/+	8.1	1.05–63.0	8.1
Distant metastasis	−/+	54.2	<0.0001	+	TNM classification	I/II·III	0.9	0.13–5.94	0.8
TNM classification	I/II·III	29.7	<0.0001	II·III					
Progression-free survival					Progression-free survival				
S100A4 score	2/3	5.63	0.01	High score	S100A4 score	2/3	1.3	0.60–2.65	0.5
Age (years)	59/60	0.32	0.5		FIGO stage	I/II·III·V	0.5	0.11–2.29	0.3
FIGO stage	I/II·III·V	36.8	<0.0001	II·III·IV	LN metastasis	−/+	2.7	0.94–7.63	0.07
LN metastasis	−/+	19.0	<0.0001	+	Distant metastasis	−/+	37.3	2.9–480.6	0.006
Distant metastasis	−/+	67.1	<0.0001	+	TNM classification	I/II·III	0.6	0.18–2.30	0.4
TNM classification	I/II·III	33.2	<0.0001	II·III					
LN, lymph node									

## Data Availability

The data sets generated during and/or analyzed during the current study are available from the corresponding author on reasonable request.

## Supplementary Materials

The following supporting information can be downloaded at: https://www.mdpi.com/article/10.3390/cancers17020184/s1, Figure S1: dot plot analyses for S100A4 IHC scores in 120 OCCC cases. Based on the cutoff values, cases are divided into high and low expression categories; Figure S2: (A) Western blot analysis for the indicated proteins in total lysates with the indicated protein volumes from TOV-21G (TOV) and OVISE (OV) cell lines. (B) Western blot analysis for the indicated proteins in total lysates from S100A4 OE and mock cells (Mo). (C) Left: Western blot analysis for the indicated proteins in total lysates from S100A4 KO and parental cells (P). Right: sequence analysis for S100A4 gene in parental (upper) and KO#17 cell lines (lower); Figure S3: original images of Western blot analysis for the indicated proteins in total lysates from S100A4 KO and parental cells following re-stimulation of serum-starved (24 h) cells with 10% serum (A) and 40 μM CDDP treatment (B). The reconstructed images of all blots with membrane edges visible are shown because some of the original full-length blots were cut prior to hybridization with antibodies; Figure S4: original images of Western blot analysis for the indicated proteins in total lysates from S100A4 OE and mock cells following re-stimulation of serum-starved (24 h) cells with 10% serum (A) and 10 μM CDDP treatment (B). The reconstructed images of all blots with membrane edges visible are shown because some of the original full-length blots were cut prior to hybridization with antibodies; Figure S5: interaction between S100A4 and p53 in OCCC cells. TOV-21G cell lysates were subjected to immunoprecipitation (IP) with the indicated antibodies, before Western blotting (WB) for p53 and MYH9 proteins. Input was 5% of the total cell extract. Normal rabbit IgG was used as a negative control and PARP1 was used as a positive control; Figure S6: original images of Western blot analysis for the indicated proteins in total lysates from S100A4 KO and parental cells (A) and S100A4 OE and mock cells (B). The reconstructed images of all blots with membrane edges visible are shown because some of the original full-length blots were cut prior to hybridization with antibodies; Table S1: Correlation between S100A4 expression and clinicopathological factors in ovarian clear cell carcinoma.
