# Supplementary material for: Prognostic Significance of S100A4 in Ovarian Clear Cell Carcinoma: Its Relation to Tumor Progression and Chemoresistance
Source: Cancers (Basel). 2025 Jan 8;17(2):184. doi: 10.3390/cancers17020184 (PMC11763377; doi:10.3390/cancers17020184)
Supplement: Supplementary file 1 [file cancers-17-00184-s001.zip › Supplementary Table S1.pdf]

Supplementary Table S1. Correlation between S100A4 expression and clinicopathological factors in ovarian clear cell carcinoma

		S100A4 score		
		High (≧3)	Low (<3)	
	n	n (%)	n (%)	*P*-value
Age (years)	49	25 (51)	24 (49)	
≧ 60	71	32 (45)	39 (55)	0.5
< 60				
FIGO stage				
I	71	23 (32.4)	48 (6736)	
II/III/IV	49	34 (69.4)	15 (30.6)	< 0.0001
pT				
pT1	77	25 (35)	50 (65)	
pT2/pT3	43	30 (69.7)	13 (30.3)	0.0003
LN metastasis				
Positive	15	10 (66.7)	5 (33.3)	
Negative	87	37 (42.5)	50 (57.5)	0.08
Distant metastasis				
Positive	4	4 (100)	0 (0)	
Negative	115	52 (45.2)	63 (54.8)	0.03
Dissemination				
Positive	30	21 (70)	9 (30)	
Negative	87	34 (39)	53 (61)	0.003

LN, lymph node
pT factor refers to TNM classification.
