# Supplementary figures and images for: Prognostic Significance of S100A4 in Ovarian Clear Cell Carcinoma: Its Relation to Tumor Progression and Chemoresistance
Source: Cancers (Basel). 2025 Jan 8;17(2):184. doi: 10.3390/cancers17020184 (PMC11763377; doi:10.3390/cancers17020184)

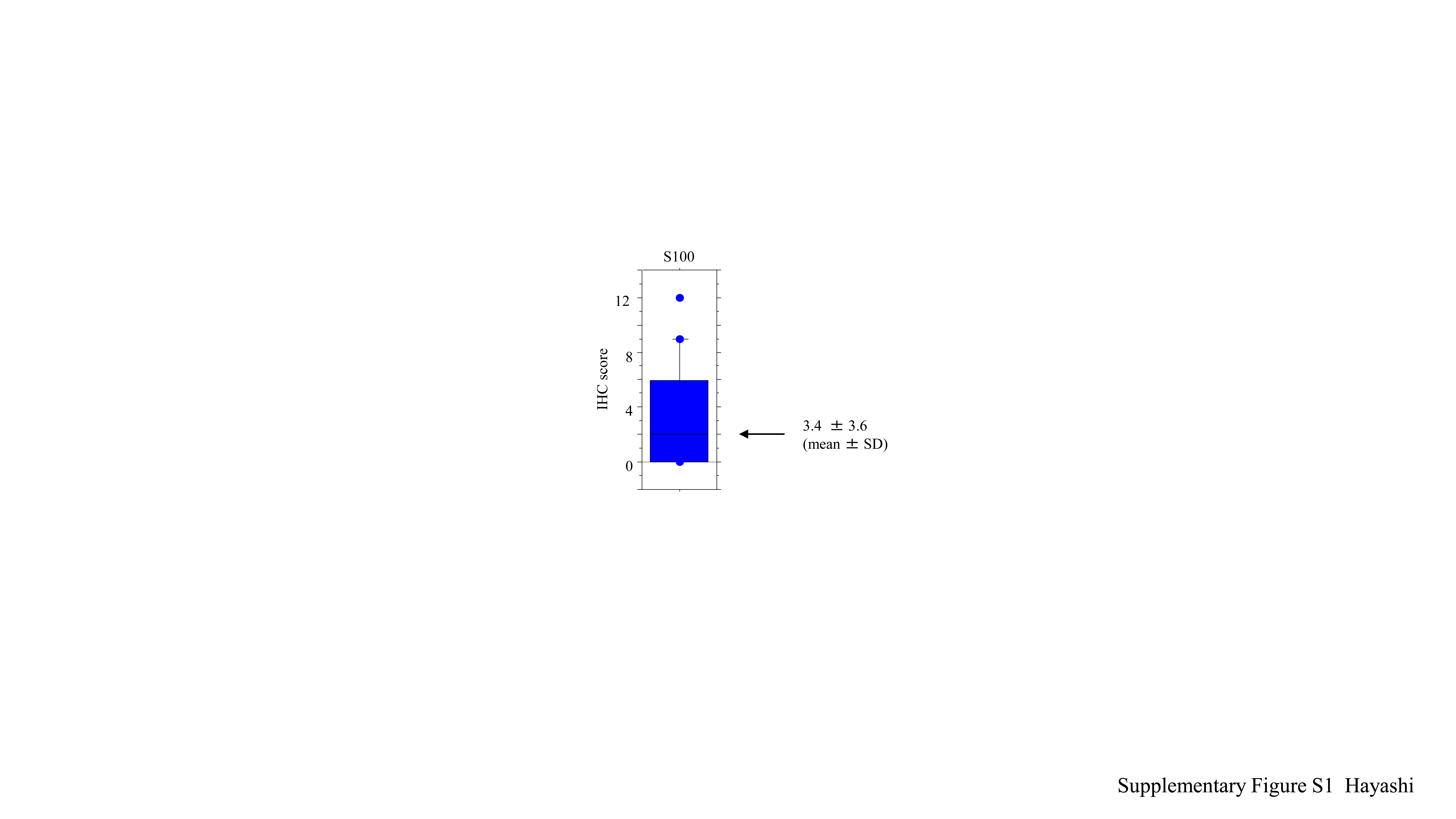

Supplement: Supplementary file 1 [file cancers-17-00184-s001.zip › Supplementary Figure S1.tif]

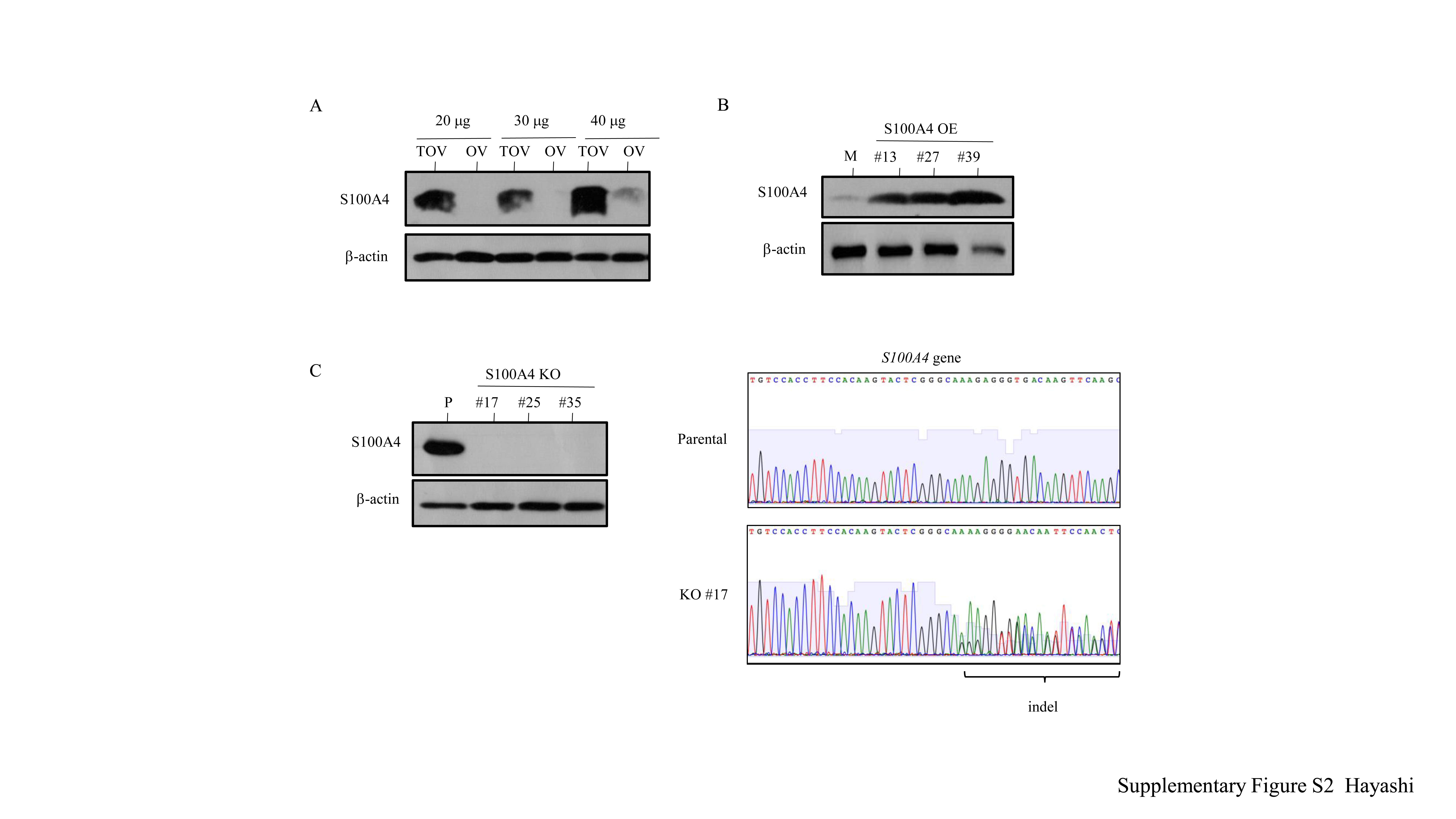

Supplement: Supplementary file 1 [file cancers-17-00184-s001.zip › Supplementary Figure S2.tif]

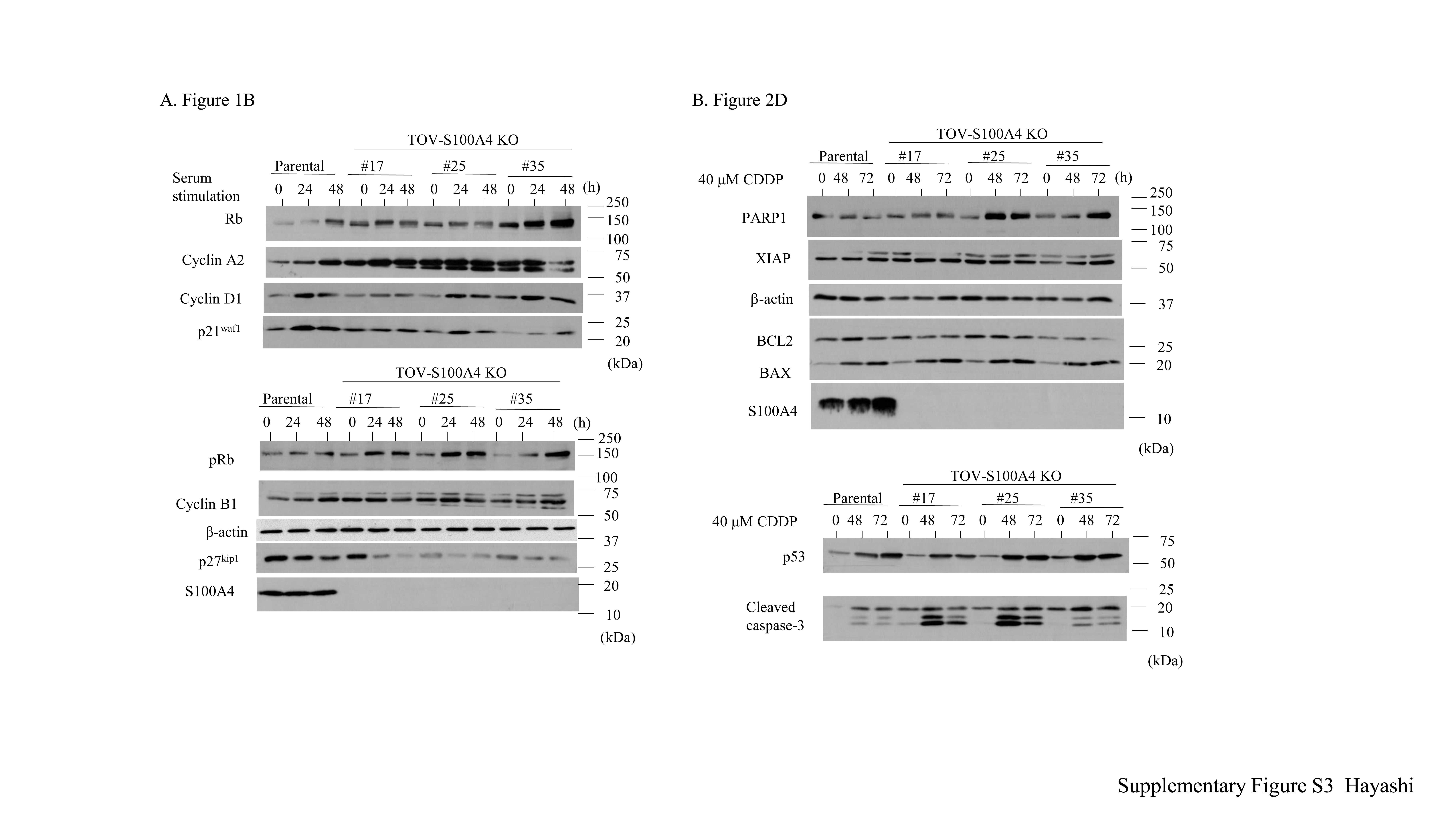

Supplement: Supplementary file 1 [file cancers-17-00184-s001.zip › Supplementary Figure S3.tif]

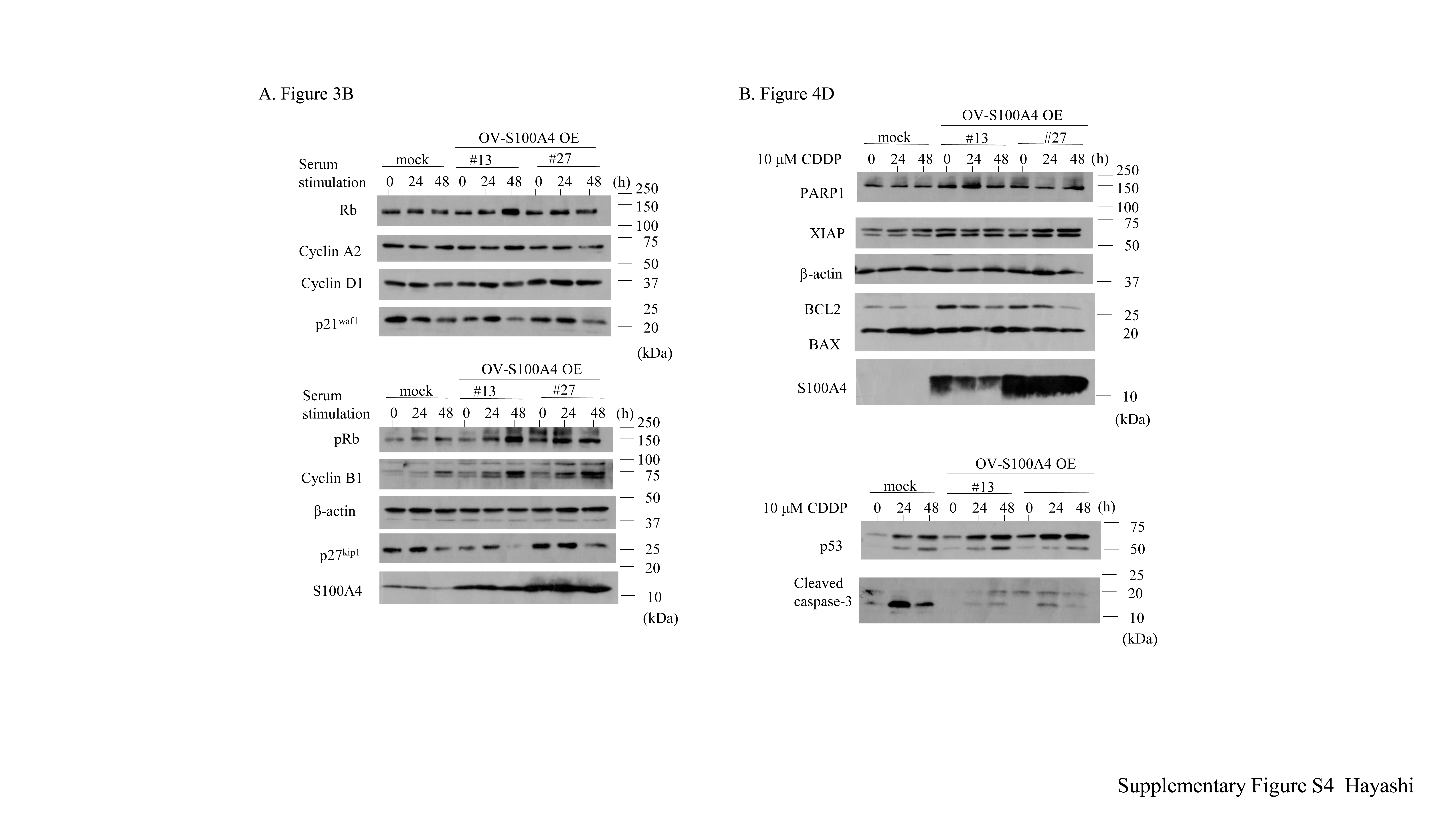

Supplement: Supplementary file 1 [file cancers-17-00184-s001.zip › Supplementary Figure S4.tif]

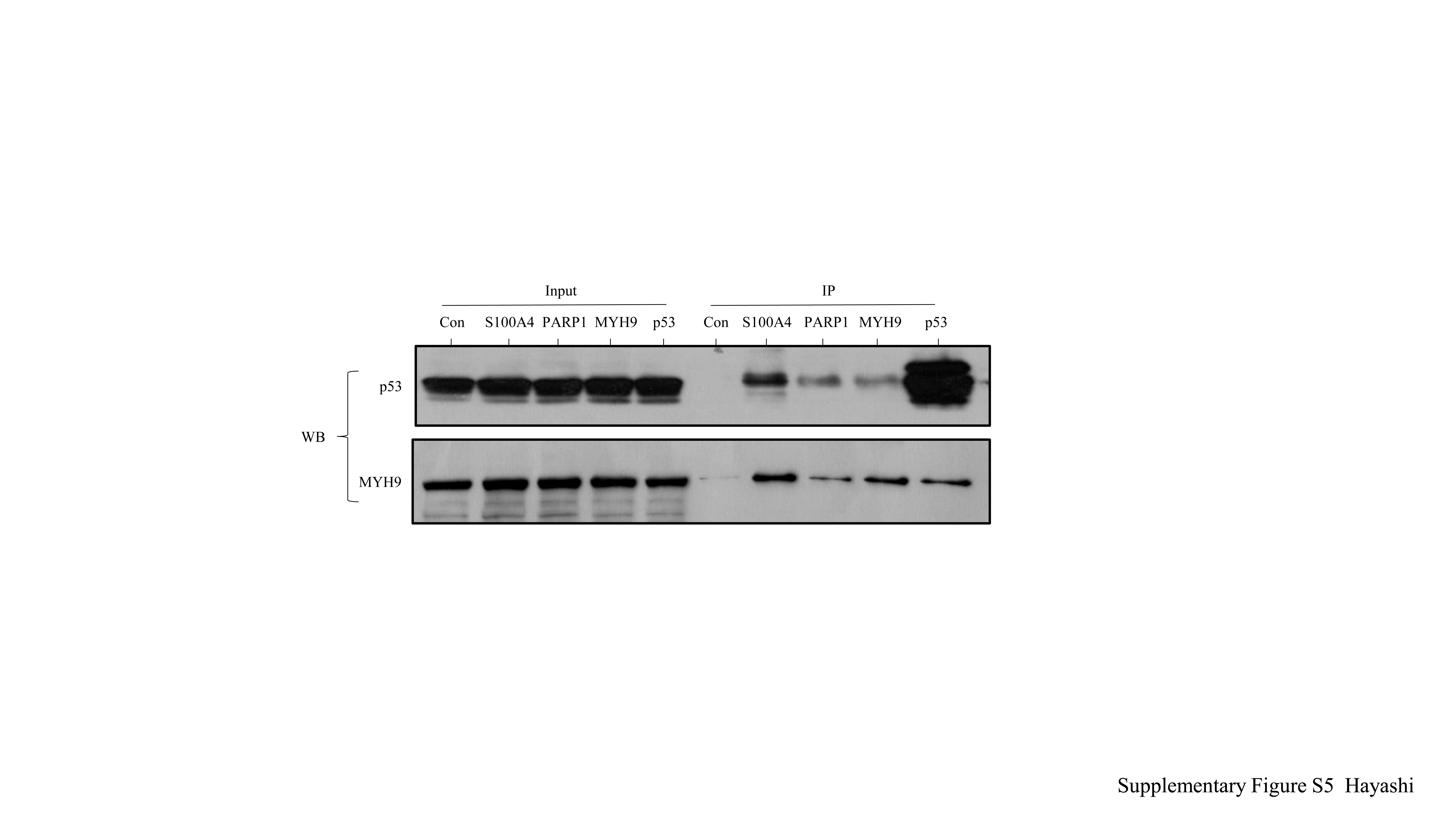

Supplement: Supplementary file 1 [file cancers-17-00184-s001.zip › Supplementary Figure S5.tif]

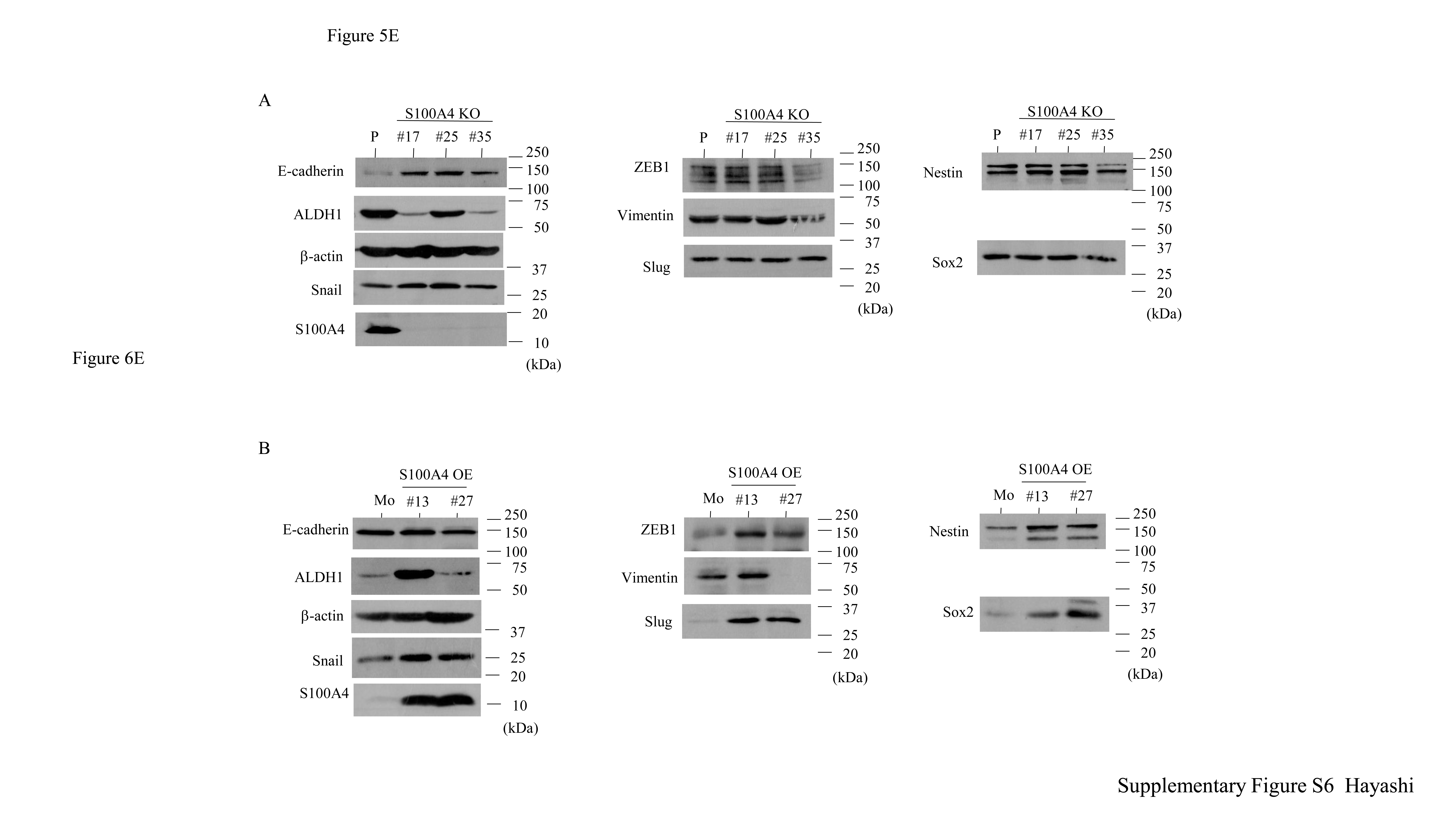

Supplement: Supplementary file 1 [file cancers-17-00184-s001.zip › Supplementary Figure S6.tif]
